# Nature-Based Tourism Elicits a Phenotypic Shift in the Coping Abilities of Fish

**DOI:** 10.3389/fphys.2018.00013

**Published:** 2018-02-05

**Authors:** Benjamin Geffroy, Bastien Sadoul, Amine Bouchareb, Sylvain Prigent, Jean-Paul Bourdineaud, Maria Gonzalez-Rey, Rosana N. Morais, Maritana Mela, Lucélia Nobre Carvalho, Eduardo Bessa

**Affiliations:** ^1^Center of Study of the Meridional Amazon, Federal University of Mato Grosso, Sinop, Brazil; ^2^Institute of Biological, Human and Social Sciences, Federal University of Mato Grosso UFMT, Sinop, Brazil; ^3^Ifremer, IRD, Centre National de la Recherche Scientifique, UMR MARBEC, University of Montpellier, Palavas-Les-Flots, France; ^4^Environmental Physiology and Toxicology, Department of Biological Sciences, University of Calgary, Calgary, AB, Canada; ^5^Wellcome Trust Centre for Human Genetics, University of Oxford, Oxford, United Kingdom; ^6^Biogenouest, Biosit - UMS Centre National de la Recherche Scientifique 3480/US INSERM 018, University of Rennes 1, Rennes, France; ^7^Centre National de la Recherche Scientifique, UMR 5805, Aquatic Toxicology, University of Bordeaux, Arcachon, France; ^8^Department of Cellular Biology and Physiology, Federal University of Paraná, Curitiba, Brazil; ^9^Graduate Program in Ecology, University of Brasília, Distrito Federal, Brazil

**Keywords:** coping style, ecotourism, conservation, behavior, gene expression, cortisol, neurogenesis, fish

## Abstract

Nature-based tourism is gaining extensive popularity, increasing the intensity and frequency of human-wildlife contacts. As a consequence, behavioral and physiological alterations were observed in most exposed animals. However, while the majority of these studies investigated the effects of punctual exposure to tourists, the consequences of constant exposition to humans in the wild remains overlooked. This is an important gap considering the exponential interest for recreational outdoor activities. To infer long-term effects of intensive tourism, we capitalized on *Odontostilbe pequira*, a short-lived sedentary Tetra fish who spends its life close to humans, on which it feeds on dead skin. Hence, those fish are constantly exposed to tourists throughout their lifecycle. Here we provide an integrated picture of the whole phenomenon by investigating, for the first time, the expression of genes involved in stress response and neurogenesis, as well as behavioral and hormonal responses of animals consistently exposed to tourists. Gene expression of the mineralocorticoid (and cortisol) receptor (*mr*) and the neurogenic differentiation factor (*NeuroD*) were significantly higher in fish sampled in the touristic zone compared to those sampled in the control zone. Additionally, after a simulated stress in artificial and controlled conditions, those fish previously exposed to visitors produced more cortisol and presented increased behavioral signs of stress compared to their non-exposed conspecifics. Overall, nature-based tourism appeared to shift selection pressures, favoring a sensitive phenotype that does not thrive under natural conditions. The ecological implications of this change in coping style remain, nevertheless, an open question.

## Introduction

Humans are currently occupying all continents, generating different sources of threats to other species, also known as human-induced rapid environmental change (HIREC) (Sih, 2013). Most of the HIREC are linked to extensive harvesting (Darimont et al., 2015), pollution (Browne et al., 2015), habitat fragmentation (Wilson et al., 2015), exotic species introduction (Tófoli et al., 2016) and climate change (Thomas et al., 2004). Recently, increasing attention has been drawn toward tourism, especially nature-based tourism, which has gained exponential popularity (Blumstein et al., 2017). With more than 8 billion visitors in terrestrial protected areas per year (Balmford et al., 2015), this tourism could be considered as a new source of HIREC.

Several deleterious effects accompanying nature-based tourism were reported worldwide, such as pest transmission (Köndgen et al., 2008), habitat destruction (Ballantyne et al., 2014) or exotic species introduction (Anderson et al., 2015). But nature-based tourism is also suggested to directly affect animals' wellbeing in a negative way (Cressey, 2014). Although all benign human-wildlife interactions do not necessarily result in such dramatic negative events, they almost always lead to small or unnoticeable, changes in animal behavior and physiology (Geffroy et al., 2015, 2016). However, so far, no general pattern has emerged to characterize the effects of nature-based tourism on physiology and behavior of wildlife species (Tablado and Jenni, 2015). Indeed, while some animals display signs of habituation to human presence, characterized by reduced stress responses to human visitation, others sensitize instead (Tablado and Jenni, 2015; Geffroy et al., 2017).

At the physiological level, tourism exposure disrupts the regulation of the stress axis; i.e., the Hypothalamo-Pituary-Adrenal (HPA)/Interrenal (in fishes, HPI) axis. The HPA/HPI axis is responsible for the production of glucocorticoids (either corticosterone or cortisol, depending on the species), which are considered as the major stress hormone. Some studies showed an increased sensitivity of the axis, with stronger production of cortisol (Walker et al., 2005; Ellenberg et al., 2007; Lima et al., 2014) and others a decreased response of the axis (Fowler, 1999; Walker et al., 2006; Villanueva et al., 2011). Similarly, at the behavioral level, some authors describe habituation to human presence, demonstrated for instance by reduced flight initiation distance, while others show increased alarm responses during human visitation (Geffroy et al., 2017).

Main reasons evoked to explain these inconsistencies in responses to tourism are differences in species, types of stressor and age, but most probably intensities of tourism exposure (Bessa and Gonçalves-de-Freitas, 2014; Geffroy et al., 2015). However, no clear information has been gathered in the context of intense exposure, or chronic exposure to tourism, despite the expected boom of ecotourism in the coming years (Balmford et al., 2015). Whether chronic exposure to tourism is assimilated by wildlife species as a stimulating (e.g., enriched environment), a monotonous, or stressful environment still needs to be described. Fish are good models to investigate these points as they generally tolerate close approach, particularly when fed (reviewed in Bessa et al., 2017a). To date, most studies focused on anti-predator behavior (using e.g., Flight Initiation Distance) or change in activity pattern of fish (Bessa et al., 2017a). As an example, we recently demonstrated in 11 different fish species that behavior (i.e., diurnal activity) was modulated in response to chronic tourists' presence: they avoided massive human presence and this was consistent over various rivers within a regional range (Bessa et al., 2017b). In a different study (Bessa and Gonçalves-de-Freitas, 2014) we also demonstrated how tourism reduces territorial aggressiveness and nesting in a pike cichlid, which can impair reproduction and affect fish populations. Nevertheless, much less is known on factors, particularly mechanistic, allowing fish to cope with tourism presence. In the present study, we focused on one river to investigate the in-depth effects of eco-tourism on one fish species, by describing effects from the molecular to the population level. We considered a sedentary and opportunistic species (Tondato et al., 2013b), the Tetra fish (*Odontostilbe pequira*), which, in tourist-visited areas, lives throughout its entire life cycle in close vicinity with visitors. Moreover, the sedentary life style and the short life cycle of this species (Tondato et al., 2013b), although not representative of many other species from tourism areas, is ideal to evaluate the effect over several generations. In combination with behavioral markers, the sensitivity of the stress axis was investigated using cortisol released in the water, whereas neurogenesis was estimated with expression of the *proliferating cell nuclear antigen* (*pcna*; cell proliferation/cell cycle control) and the *neuronal differentiation factor* (*neurod*; neuronal determination and differentiation; involved in the regulation of neuronal survival and maturation). Both, *neurod* and *pcna* have been successfully used as proxies of neural plasticity and cognitive abilities of animals, including fish (Sørensen et al., 2013) and are thus considered suitable neurological markers. Fish behavior has indeed been linked to neural proliferation, as neurogenesis play a significant role in behavioral flexibility, learning and memory (Øverli and Sørensen, 2016).

Therefore, our objective with this experiment was to evaluate how tourism affects fish from the gene expression level to the endocrine level and then to the behavioral level. Lasting effects were evaluated by placing fish, from disturbed and undisturbed areas, in similar conditions: aquariums. We hypothesized that tourism changes the expression of genes related to stress; that cortisol secretion would be increased by tourism and that fish from tourist-visited zones would act bolder than those from control zones.

## Materials and methods

### Ethical statement

All experiments comply with the Brazilian legislation and agree with the Ethics Committee for Animal Research of the Federal University of Mato Grosso. Fish were collected under ICMBio license 2071343. We used the minimum number of fish for gene expression analysis to reach statistical significance. All the other fish used were kept for 17 days, anesthetized (eugenol 1/10 in alcohol, 0.3 ml.L^−1^) to measure the fork length. In order to not recapture those fish during the next period, they were then released in the closest river with similar touristic pressure.

### Study site

**Figure 1:**
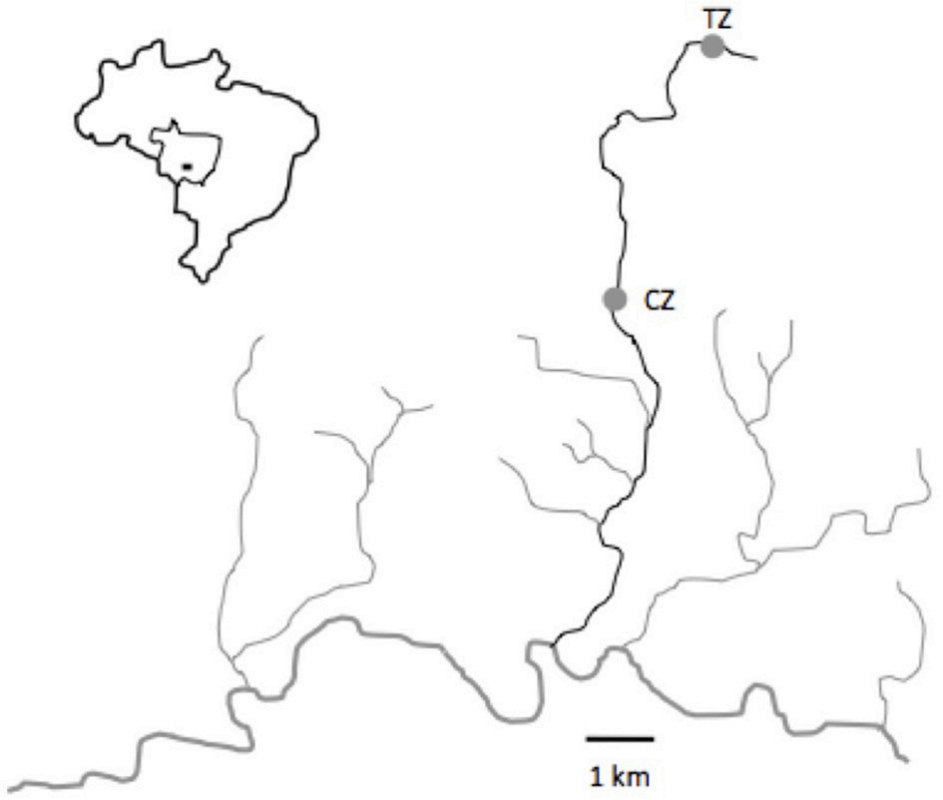
Map of the studied area in the municipality of Nobres, Mato Grosso, Brazil. TZ, Touristic zone; CZ, Control zone.

**Figure 2:**
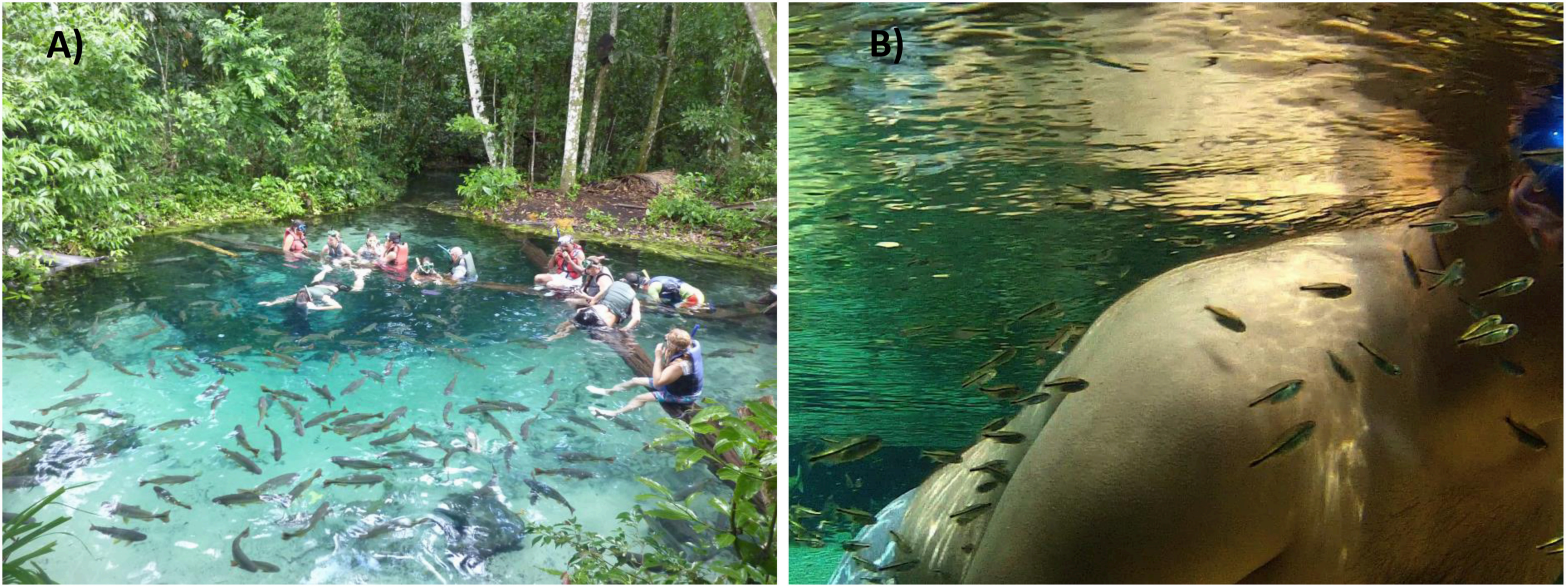
**(A)** Tourists enjoying snorkeling in clear water of nobres surrounded by fish, **(B)** *O. pequira* eating dead skin in the body of a snorkeler.

As for a previous study (Bessa et al., 2017b), fish were collected in the Cuiabazinho River Basin, at Nobres, Mato Grosso, Brazil (14° 43′ 13″S; 56° 19′ 39″W, Figure 1). Rivers around Nobres are notorious for the transparency of the water, making them a unique and remarkable place that attracts thousands of tourists every year. The tourism zone (TZ), situated in the Salobra River, has been receiving tourists for decades, with a present flow of about 15,000 tourists a year. Tourists are present daily from 9.00 a.m. to 5.00 p.m. (see Figure 2A), and are accompanied by guides that feed the fish every day. The control zone (CZ), situated 5 km downstream, does not receive any tourists. Environmental characteristics are similar throughout the river (Bessa and Gonçalves-de-Freitas, 2014). Since the main goal of the experiment was to deepen our understanding of the impact of ecotourism at multiple levels (not only behavior as performed before Bessa et al., 2017b), we used temporal replicates instead of location replicates (i.e., we collected fish from the same place multiple times instead of at the same time in multiple places). This allowed obtaining fish from similar environments, without bias introduced by locations and species interactions.

### Model species and collections

Here we focused on one small fish species (<35 mm), the tetra *Odontostilbe pequira* (Cheirodontinae), to investigate stress physiology, from gene expression to the behavioral response. This tetra was specifically chosen because it is sedentary (Tondato et al., 2013b), opportunistic and feeds on other fish scales, mucus (Lima et al., 2012), and nibbles gently on appendages of humans to remove dead skin (see Figure 2B). In high densities, they even performed cannibalism, by feeding on other fish body parts (lepidophagy *sensu* Lima et al., 2012). In addition, they are considered r-strategists, with a short life cycle and relatively high fecundity (Tondato et al., 2013a,b).

Overall, these life-history traits make them particularly suitable for studying responses to tourism, since (1) they might have readily been habituated to human presence for several generations, (2) they are philopatric, which enables us to consider that no migrations affected our results, and (3) they have disproportionally large impact on the distribution of other fish communities (Balduino et al., 2017), and could thus be considered a keystone species.

About a hundred fish from each zone were collected using hand seines in the morning (between 9.00 and 11.00 h, to avoid circadian variation) in June 4th and August 1st, 2014. Between four and six fish were euthanized at each sampling month and per condition (tourism zone, TZ, and control zone, CZ) for gene expression analysis (an additional sampling for this purpose only was conducted in July 12th, so that there are three time samples), for a total of 15 fish per condition. Fish were directly flash frozen in liquid nitrogen (an ethically accepted procedure for euthanasia of small animals) and kept at −80°C in the laboratory of the Federal University of Mato Grosso, before being shipped to Bordeaux and Rennes, France, for gene expression measurements in brain. Remaining fish were brought alive to the laboratory for further stress related measurement (see below). Underwater monitoring of fish density was conducted on the 10th of August and the 28th of September 2014. Most Tetras were occupying river boarders and by recording throughout a 34 m length transect in the CZ and a 46 m length transect in the TZ it was possible to assess the mean number of fish in the area. The first 30 cm below the surface were video recorded, and fish density (number/m^2^) was extrapolated from fish number assessment. In August, 2.1 fish/m^2^ were detected in the CZ and 36.6 fish/m^2^ in the TZ. In September, 2 fish/m^2^ were detected in the CZ and 30.5 fish/m^2^ in the TZ.

### Gene expression analysis

Here we focused on brain gene expression of the two well-described receptors of cortisol—GR and MR– involved in the HPI axis regulation. We also investigated markers of cell proliferative activity and neural differentiation and survival of new born neurons, respectively PCNA and NeuroD (Sørensen et al., 2013). *Odontostilbe pequira* were measured (standard body length: 2.5 ± 0.5 cm; weight: 217 ± 122 mg) and dissected in France. The brain was placed in RNAlater® solution for 1 day and stocked at −80°C prior to analysis.

### Production of primers for the selected genes, extraction, reverse transcription and quantification of cDNA

Total RNAs were extracted from the tissues using the SV Total RNA isolation System kit (Promega®), following manufacturer's guidelines. Moreover, to maximize lipid and protein elimination, a step of phenol–chloroform– isoamylic alcohol (25:24:1) extraction was added as reported in Geffroy et al. (2012). Then, 0.5 μg of RNA from the brain was reverse transcribed using 200U Moloney murine Leukemia virus (MMLV) reverse transcriptase (Promega) and 2 μg random hexamers (Promega) in a master mix buffer and supplemented with 25U of RNase inhibitor (RNasin; Promega) in a final volume of 25 μl. Reverse transcription products were then diluted 1:10 for quantitative real-time PCR (qPCR).

**Figure 3:**
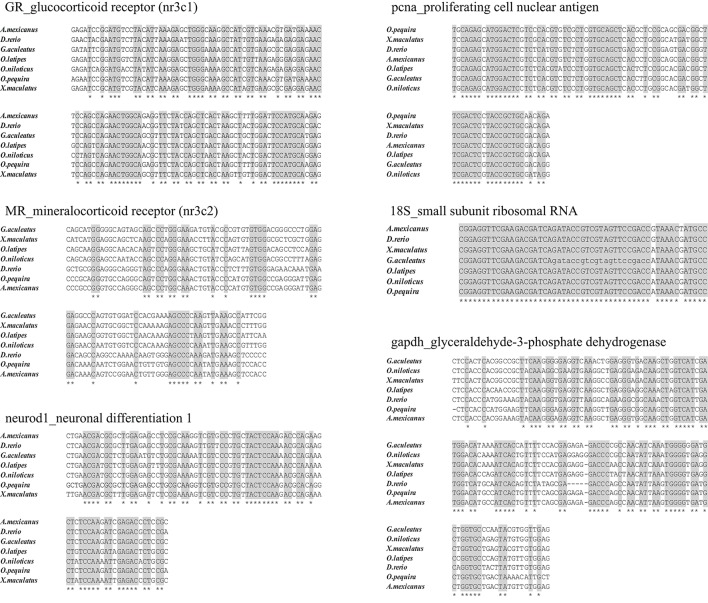
Alignment of the conserved sequences in the teleosts (stickleback, tilapia, platy, medaka, danio rerio, and cave fish). The homology search with sequence of *O. pequira* was carried out with BlastSearch of Ensembl. Multiple sequences alignment was performed using EBI Clustal Omega promgram. Conserved Nucleotides are indicated in gray with stars. Our sequence data are available in GeneBank databases under the accession numbers: KU820855 (gapdh), KU820856- KU820857 (GR-MR respectively), KU820859-KU820860 (neurod1-pcna respectively), and KU820862 (18S).

**Figure 4:**
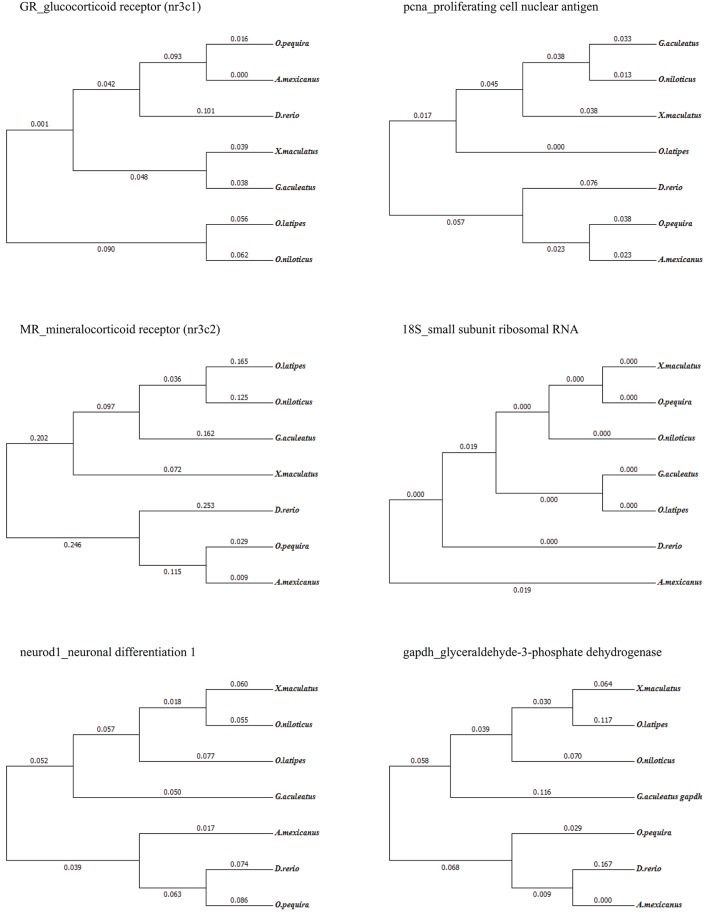
A phylogenetic tree derived from the multiple sequence alignment of *O. pequira* relative to existing and described sequences in other fish species. Phylogenetic analysis was constructed with the maximum likelihood methodby MEGA (version 7.0.14) program based on gene nucleotide sequences from various fish species.

We took advantage of the recent sequencing of a phylogenetically close species (*Astyanax mexicanus*) of the same family (Characidae) and chose highly conserved regions of other available fish species *Xiphophorus maculatus* and *Danio rerio* on ENSEMBL to align sequences (ClustalW multiple alignment) and design primers for selected genes (primer3). All primers used in this study are listed in Table 1. We then sequenced amplicons and verified that partial cDNA sequences corresponded to the correct gene (Figure 3, 4). The phylogenetic analysis was constructed with the maximum likelihood method by MEGA (version 7.0.14). These sequences were deposited in NCBI.

**Table 1:** Primer sequences for all genes tested (available in NCBI).

**Gene**	**GenBank accession numbers**	**Primers**	**Primer sequence**
gapdh	KU820855	gapdh-F	5′CAATGACCCCTTCATTGACC3′
		gapdh-R	5′TAGTCAGCACCAGCATCACC3′
gr	KU820856	gr-F	5′GGAACACGCAGCACTATGTC3′
		gr-R	5′CCTCCCGACTGTTTTCATGT3′
mr	KU820857	mr-F	5′TGAGTCCATGGGCATCTACA3′
		mr-R	5′ATGGTGTTGGTGGAGCTTTC3′
neurod1	KU820859	neurod1-F	5′AGATGCGGCGCATGAAGGCGAACGC3′
		neurod1-R	5′CGGAGSGTCTCGATCTTGGAGAGCT3′
pcna	KU820860	pcna-F	5′GACCTGATCACCGAGGCYTGCTGGG3′
		pcna-R	5′CTGTCGCAGCGGTAGGAGTCG3′
18S	KU820862	18S-F	5′TCGCTAGTTGGCATCGTTTAT3′
		18S-R	5′CGGAGGTTCGAAGACGATCA3′

RNA abundance analysis of genes encoding GR, MR, Neurod, and PCNA in the brain was done using qPCR. Concerning GR, the isoform amplified was within the GR2 cluster. Note that it is unknown whether *O. pequira* has the two isoforms, but other fish species such as the zebrafish and the spotted gar only have one. Samples of cDNA were analyzed using the GoTaq® qPCR Master Mix (Promega) following manufacturer instructions (details of the qPCR protocol are provided in the Supplementary Material). Glyceraldehyde-3-phosphate dehydrogenase (*gapdh*) and ribosomal 18S (*18S*) were used as housekeeping genes (Goidin et al., 2001; Bland et al., 2012). For each gene, standard quantity was automatically calculated using the linear dynamic range of each target (StepOneTM Software v2.3). The mean of two experimental duplicates was then divided by the geometric mean of housekeeping genes (*gapdh* and *18S*). Neither *gapdh* nor *18S* significantly differed (*n* = 27; gapdh *p* = 0.55; 18S *p* = 0.28) between the two conditions (TZ and CZ).

### Behavioral and endocrine measure in laboratory conditions

In June (the 4th), we distributed 136 fish in eight aquaria (17 fish per aquaria and four aquaria per condition: TZ and CZ), provided with the water from the river. The number of fish constituting the group was chosen for three main reasons, (i) ensuring fish well-being by providing numerous individuals for this gregarious species, (ii) having sufficient number of individuals to detect cortisol in the water (iii) without having them too crowded in 30 L aquaria. Aquaria were visually isolated and filmed from above (gopro3 HD black edition) once a day for 20 min. Fish were fed commercial diet for tropical fish (Poytara) once a day, from the second day after the transfer to the aquaria without visual contact with the feeder. All fish started to feed from day 2. Aquaria received artificial lighting (MOSAICTM, 9.6 W: 100–240 W) in a 12 h/12 h dark-light cycle respecting the natural daylight time. Four times a day (9:00, 11:00, 13:00, and 17:00), 35% of the water was renewed automatically by letting open flow water from a river pass through the tanks for 30 min. This water used in renewals does not come directly from tourism areas or from either tourism or control zones. This ensured nitrites (measured by Nitrite Labcon Test) were constantly at 0 ppm.

The same procedure was repeated on the 1st of August with 204 fish placed in 12 aquaria (17 fish per aquaria and six aquaria per condition: TZ and CZ).

At each trial (June and August), fish were left to acclimate for 12 days in their new environment. Temperature was monitored four times per day, just before renewing water. Mean temperature was 26.3 and 26°C respectively in June and August.

After 12 days of acclimation, fish were submitted to mechanical stress to evaluate whether fish responded to an unpredicted event according to whether or not they had previously been exposed to tourists. To do that we simultaneously hit the aquaria with an iron bar covered in cloth for 10 s, resulting in noise and vibration triggering a short “freezing” response. Their response was recorded 10 min before and 10 min after this stress. We analyzed speed and space use of each individual from 10 s before stress to 40 s after stress. This duration was chosen because it was sufficient to assess the basal activity level (before stress) and cover the time needed to get back to this basal level after the stress. We also analyzed space use (distance from the center of the aquarium) of each fish during the 20 min of the experiment to obtain data on the thigmotaxis propensity of each fish. Both thigmotaxis and freezing were assessed as both are recognized indicators of anxiousness and stress in fish (Kalueff et al., 2013; Schreck et al., 2016). See Supplementary Material for the full details of mathematical models of fish behavior.

On the first day of mechanical stress (in June and August), 500 ml of water of each aquarium was sampled at different time point. In order to obtain basal cortisol emitted by all fish, water was collected 1 h 20 min before stress (at 11 h 30). Ten minutes after the stress (at 13 h 00) 6 L of water were added while outflow tubes were closed (the water was completed 5 cm up to the limit). Then 500 ml from each aquarium were sampled at 0.5, 1.5, 2.5, and 3.5 h after stress. Water samples were then conserved at −20°C before being analyzed for cortisol (Ellis et al., 2004; Sadoul et al., 2015).

### Quantification of cortisol in water samples

To allow for repeated cortisol measures from this small fish and avoid manipulation stress, we collected cortisol from the water (as performed by Sadoul et al., 2015). Water samples (500 ml) were first filtered by gravity through a nylon filter (100 μm pore-size). The water was then peristaltically pumped at about 25 ml/min through a syringe filter (Millex®, 0.45 μm pore-size), which was connected to an activated solid phase extraction cartridge (Sep-pak® Plus C18, Waters Ltd., U.K.). After pumping, corticosteroids were immediately retrieved from the cartridge by elution with 5 mL of organic solvent (ethylacetate/cyclohexane, 1/1 vol/vol). Eluate was then frozen at −20°C overnight, transferred to a fresh tube to conserve only the organic phase and evaporated at 40°C. Tubes were then kept at −20° C until enzyme immunoassay measurement of cortisol. Cartridges were reused after reactivation up to 3 times, since no effects of reuse was observed (Sadoul et al., 2015).

Dry cortisol extracts were re-dissolved in 300 μl of steroid buffer (0.1 M NaPO4, 0.149 M NaCl, pH 7.0) prior to analysis. Cortisol was quantified by enzyme-immunoassay using a cortisol -horseradish peroxidase ligand (1:20,000 dilution) and the antibody for cortisol (polyclonal R4866; 1:8,500 dilution). R4866 cross-reacts with 100% cortisol, 9.9% prednisolone, 6.3% prednisone, 5% cortisone, 0.7% corticosterone, 0.3% deoxycorticosterone, and 0.5% 21-deoxycortisone. Parallel displacement curves were obtained for water extracts by comparing serial dilutions of pooled water extracts (1:1–1:16) and the cortisol standard preparation (hydrocortisone; Sigma-Aldrich, St. Louis, MO; 3.9–1,000 pg/50 μl). Samples were assayed in duplicates and intra- and inter-assay coefficients of variation were <10%. Cortisol concentrations are expressed as nanograms per liter of water per mm of fish.

### Data analysis and ethic note

#### Gene expression

Comparisons of gene expression between TZ and CZ were assessed using a linear model with mixed effects (lme, Laird and Ware, 1982) for each gene. Due to the weak number of individuals (i.e., 5 per zone) sampled at each time point (i.e., trials: June, July, and August), we pooled all individuals per zone (fixed effect) and considered trials as a random variable for the intercept such as: Gene ~ Zone + (1|Trial).

#### Cortisol

We used linear model with mixed effects (lme) to compare the cortisol production (variable response) over time (explanatory variable: fixed) during the stress protocol between zones: TZ and CZ (explanatory variable: fixed), once placed in controlled conditions. We first analyzed total cortisol production for the two trials (June and August, data pooled by condition). As repeated values of cortisol quantity were obtained over time (at 11:30, 13:30, 14:30, 15:30, and 16:30) for each aquarium, we considered the aquarium as a random effect (intercept) nested within trials and time as random for the slope of each aquarium. We compared models with additive terms (zone + time) and interaction between terms (zone x time) using the Akaike's Information Criterion (AIC) (Bozdogan, 1987) for the model with random slope and intercept, or with random intercept only, such as four models were compared:

(1) $${\text{Y}}_{\text{i},\text{t}}=(\alpha +\alpha_{\text{i}})+\beta t+\varepsilon_{\text{i},\text{t}}$$

(2) $${\text{Y}}_{\text{i},\text{t}}=(\alpha +\alpha_{\text{i}})\times \beta t+\varepsilon_{\text{i},\text{t}}$$

(3) $${\text{Y}}_{\text{i},\text{t}}=(\alpha +\alpha_{\text{i}})+(\beta +\beta_{\text{i}})t+\varepsilon_{\text{i},\text{t}}$$

(4) $${\text{Y}}_{\text{i},\text{t}}=(\alpha +\alpha_{\text{i}})\times (\beta +\beta_{\text{i}})t+\varepsilon_{\text{i},\text{t}}$$

Where

β: Fixed effects (Zone and Time)

ε_i, t_: Random error term

α_i_, βi: Random effects

**i:** items (aquarium) and **t:** (time)

Similarly, the four models were compared for cortisol production between each zone, but at each trial (June or August). The more parsimonious model, based on the AIC, was then conserved.

#### Behaviors

First, values of activity were normalized by the length of the fish in order to express activity as a movement in percentage of body length per second (% body length/s). The mean activity movement of all the fish at one time point as used for the analyses described below. Therefore, each tank is considered as a statistical unit. Overall, fish showed in both conditions a strong but transient increase in activity during the stress, followed by a freezing behavior taking few minutes to recover from. The temporal pattern of freezing and recovery was modeled using a Weibull curve as a function of time. The equation used was:

$$f(x)=y\;max-\alpha \frac{k}{\lambda }{\left(\frac{x+t_{min}}{\lambda }\right)}^{\left(k+1\right)}e^{-\;{\left(\frac{x+t_{min}}{\lambda }\right)}^{k}}$$

In each session, a fitting was performed on each tank and experimental day using method described by Pinheiro and Bates (2000). First, a non-linear least squares (NLS) estimate of the Weibull curve parameters was performed for each statistical unit with starting value (y_min_) fixed within each session. NLS estimates were used as the first arguments of a non-linear mixed model (nlme) describing activity using day, condition and interactions as fixed effects and tank as random effect. Parameters estimates of the Weibull curve were then used to extract minimum activity for each statistical unit, further analyzed using a linear mixed model. As for cortisol, four models were compared for each behavior: variable response (freezing intensity, time to freeze and thigmotaxis), with the full model [model (4)] containing random effects for the slope (time) and the intercept (aquarium) and interaction term between zone and time (both fixed explanatory variable). Model selection was done using AIC, conserving the one with the lowest score.

All statistical analyses were performed using R software 3.2.2 (R Core Team, 2013), and packages “lmerTest” (Kuznetsova et al., 2015) and “nlme” (Pinheiro et al., 2016) for linear mixed models and non-linear mixed models, respectively.

## Results

**Figure 5:**
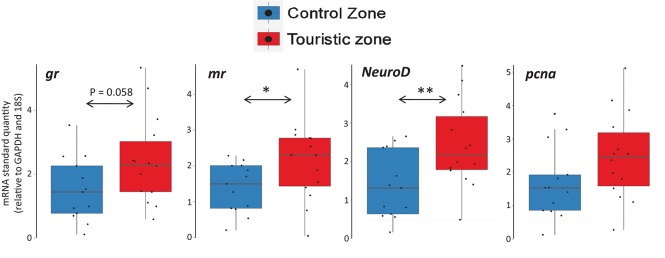
Gene expression of glucocorticoid receptor (*gr*), mineralocorticoid receptor (*mr*), *NeuroD* and *pcna*, relative to reference genes *GAPDH* and *18S*, of fish sampled in control and tourism zones. ^*^p < 0.05; ^**^p < 0.01. The central line in the box represent the median, boxes represent quartiles and whiskers maximum-minimum values.

**Figure 6:**
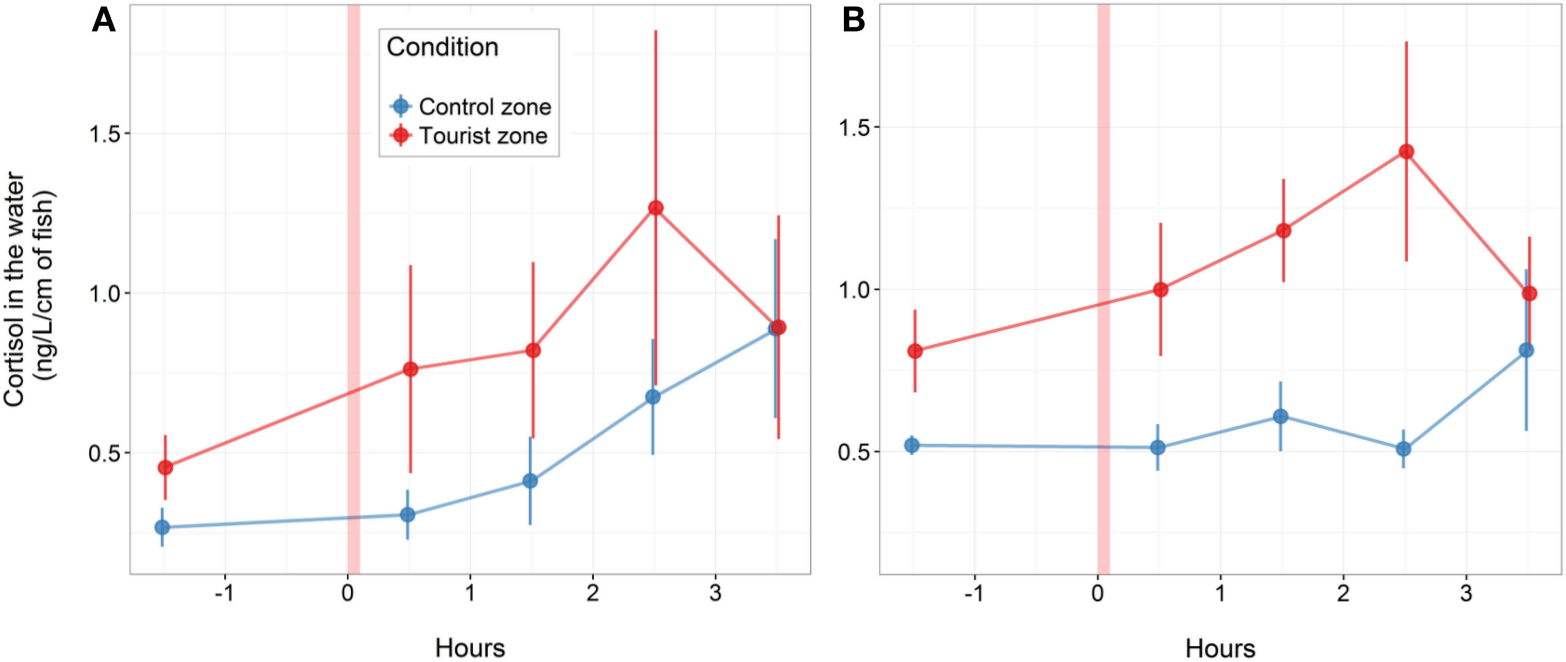
Water-borne cortisol (mean and standard error) in aquaria before and after stressor stimuli for fish from control and tourism zones. Results are shown for fish collected in June **(A)** and August **(B)** (*n* = 4 aquarium in June, *n* = 6 aquarium in August, with 17 fish per aquarium). The x-axis presents time before and after the stressor (red bar).

**Figure 7:**
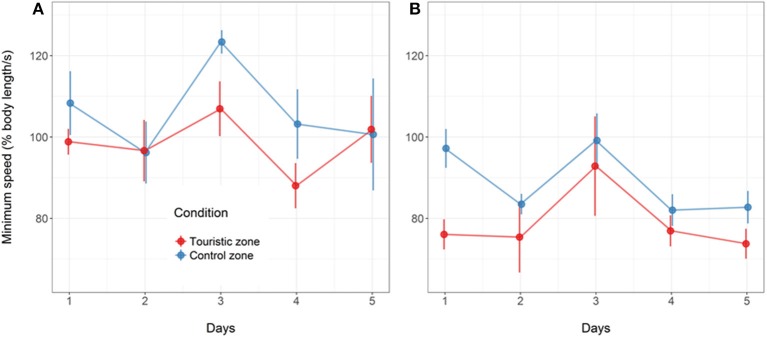
Minimum speed value (% body length.s^−1^) following artificial stress for the fish from control or tourism zones throughout the five experimental days in the two replicates of the study in June **(A)** and August **(B)**. Mean and standard error are given (*n* = 4 aquarium in June, *n* = 6 aquarium in August, with 17 fish per aquarium).

Fish from tourism and control zones differed in brain gene expression, cortisol levels in a new environment and behavioral responses to a stimulus. Tetras from the TZ displayed higher expression of genes encoding MR and NeuroD (Figure 5), higher basal and post-stress cortisol production (Figure 6) and higher propensity to freeze (Figure 7) than control zone's (CZ) fish. The fish density was highest in the tourism zone (TZ) compared to the CZ (33.5 fish/m^2^ vs. 2 fish/m^2^).

For fish directly sampled in the two locations, *mr* was upregulated in fish from the tourism zone (Figure 5, Table 2), while only a tendency was detected for *gr*. An increase in *NeuroD* was also observed in the brain of fish caught in the tourism zones, while no increase of *pcna* was noted (Figure 5, Table 2).

**Table 2:** Summary of the number of fish used at each trial (June, July, and August) for investigating gene expression in the Brain, in the two different zones (Touristic Zone, TZ and Control Zone, CZ).

	**TZ**			**CZ**			**Total TZ**	**Total CZ**	***F*-value**	***p*-value**	TZ^*^
	**June**	**July**	**August**	**June**	**July**	**August**					
Geometric Mean (GAPDH, 18S)	4(5)	6	4	6	3(5)	4(5)	14	13	0.033	0.85	=
GR	4(5)	6	4	6	3(5)	4(5)	14	13	3.95	0.058	+
MR	4(5)	6	4	6	3(5)	4(5)	14	13	4.68	0.04	+
NeuroD	4(5)	6	4	6	3(5)	4(5)	14	13	8.69	0.007	+
PCNA	4(5)	6	4	6	3(5)	4(5)	14	13	2.85	0.10	=

*Numbers in bracket correspond to the total number of fish sampled in the zone, since a total of 3 fish were not used for technical reasons. The output of the linear model with mixed effects (lme) is given for each gene, with the final column indicating the direction of the effect (TZ: “+” indicates that the gene is more expressed in fish from the TZ zone compared to those from the CZ)*.

After being placed in a novel environment (aquariums) for 12 days, cortisol production increased similarly between fish from tourism and fish from control zone over the 3.5 h following the stress (the interaction time × zone was not significant). Fish from the tourism zone, however, produced significantly more cortisol than fish from the control zone before [basal, *t*-value: 4.1 and p: 0.025, model (i), Table 3] and after the artificial stress [*t*-value: 3.12 and p: 0.005, model (i), Table 3, Figure 6]. This was observed independently over two time replicates; in June with a tendency [*t*-value: 2.02 and p: 0.066, model (ii), Table 3] and in August [*t*-value: 4.4 and *p* < 0.001, model (iii), Table 3].

**Table 3:** Outputs of different linear mixed model testing the effects of time and zone (touristic or control) on cortisol production for both trial [model (i)] and at each trial [model (ii), model (iii)].

**Model**	**Cortisol** ~ **Zone** + **Time** + **(1|Trial/Aquarium)**					
**(i) June** + **August**	**Random effects**					
		**Groups**	**Name**	**Variance**	**Std. Dev**.	***n***
		Aquarium:Trial	(Intercept)	0.055	0.23	20
		SES	(Intercept)	0.004	0.06	2
		Residual		0.17	0.41	
		Number of observations: 100				
	**Fixed effects**					
		**Estimate**	**Std error**	***t*****-value**	***p*****-value**	
	(Intercept)	0.45	0.11	4.1	0.025	^*^
	Zone:Touristic	0.42	0.13	3.2	0.005	^**^
	Time	0.08	0.024	3.5	<0.001	^***^
	**Cortisol** ~ **Zone** + **Time** + **(1**+**Time|Aquarium)**					
**(ii) June**	**Random effects**					
		**Groups**	**Name**	**Variance**	**Std. Dev**.	***N***
		Aquarium	(Intercept)	0.07	0.26	8
		Time	(Slope)	0.01	0.12	
		Residual		0.07	0.27	
		Number of observations: 40				
	**Fixed effects**					
		**Estimate**	**Std error**	***t*****-value**	***p*****-value**	
	(Intercept)	0.34	0.14	2.5	0.03	^*^
	Zone:Touristic	0.34	0.17	2.02	0.066	.
	Time	0.12	0.05	2.5	0.038	^*^
	**Cortisol** ~ **Zone** + **Time** + **(1|Aquarium)**					
**(iii) August**	**Random effects**					
		**Groups**	**Name**	**Variance**	**Std. Dev**.	***n***
		Aquarium	(Intercept)	0	0	12
		Residual		0.18	0.43	
		Number of observation: 60				
	**Fixed effects**					
		**Estimate**	**Std error**	***t*****-value**	***p*****-value**	
	(Intercept)	0.52	0.09	5.8	<0.001	^***^
	Zone:Touristic	0.49	0.11	4.4	<0.001	^***^
	Time	0.06	0.03	1.8	0.079	.

*Models with additive terms (zone + time) and interaction between terms (zone x time) were first compared using the Akaike's Information Criterion (AIC) so that the most parsimonious model was conserved*.

*** *< 0.001*,

** *< 0.01*,

* *< 0.05*.

**Figure 8:**
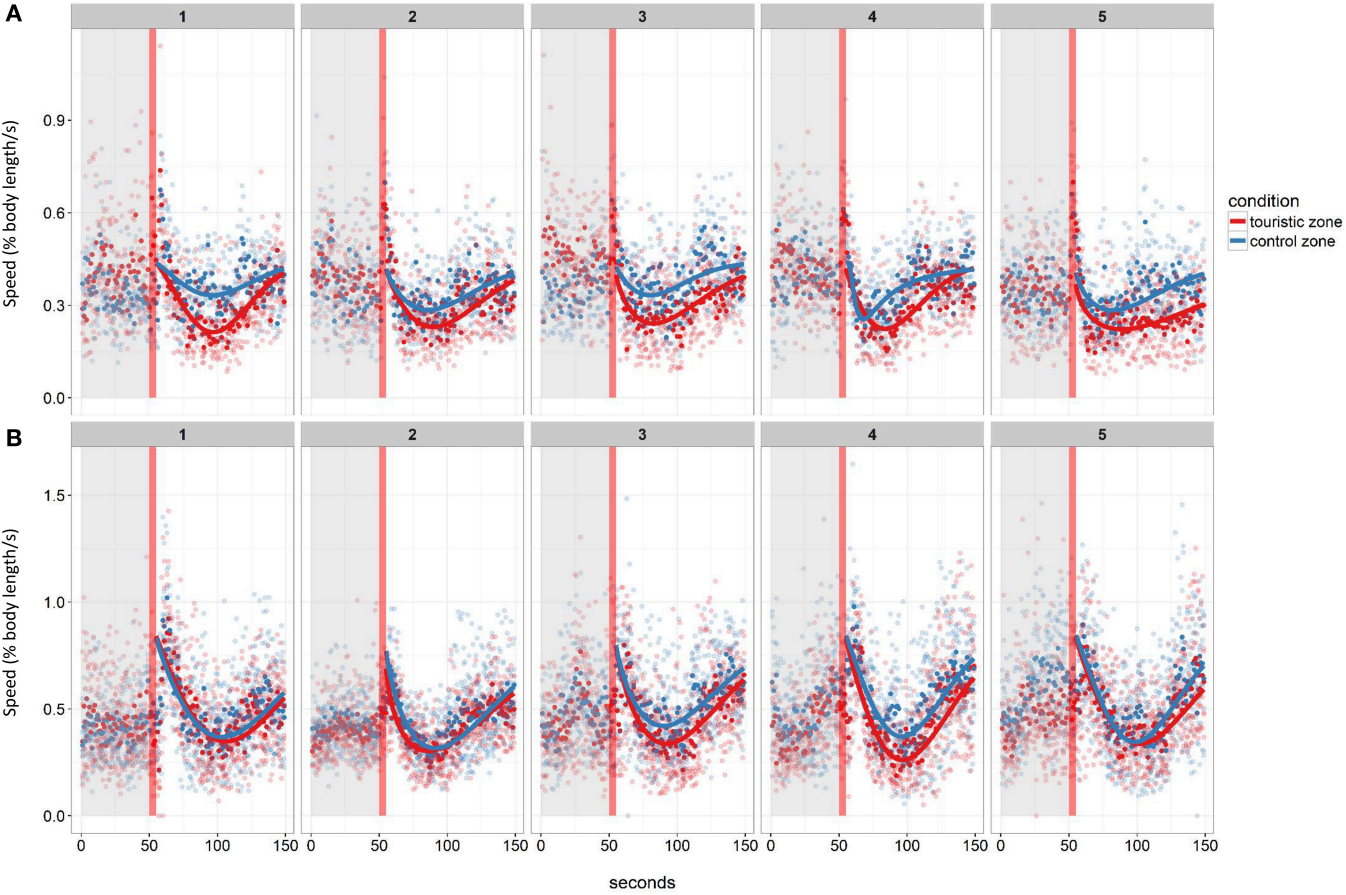
Mean swimming speed before and after the stressor (red vertical line) of fish from the control or touristic zone throughout the five experimental days in the two replicates of the study in June **(A)** and August **(B)**. Mean values per tank are given (*n* = 4 in June, *n* = 6 in August). The post-stressor period was modeled for each tank and each day using a Weibull curve.

**Figure 9:**
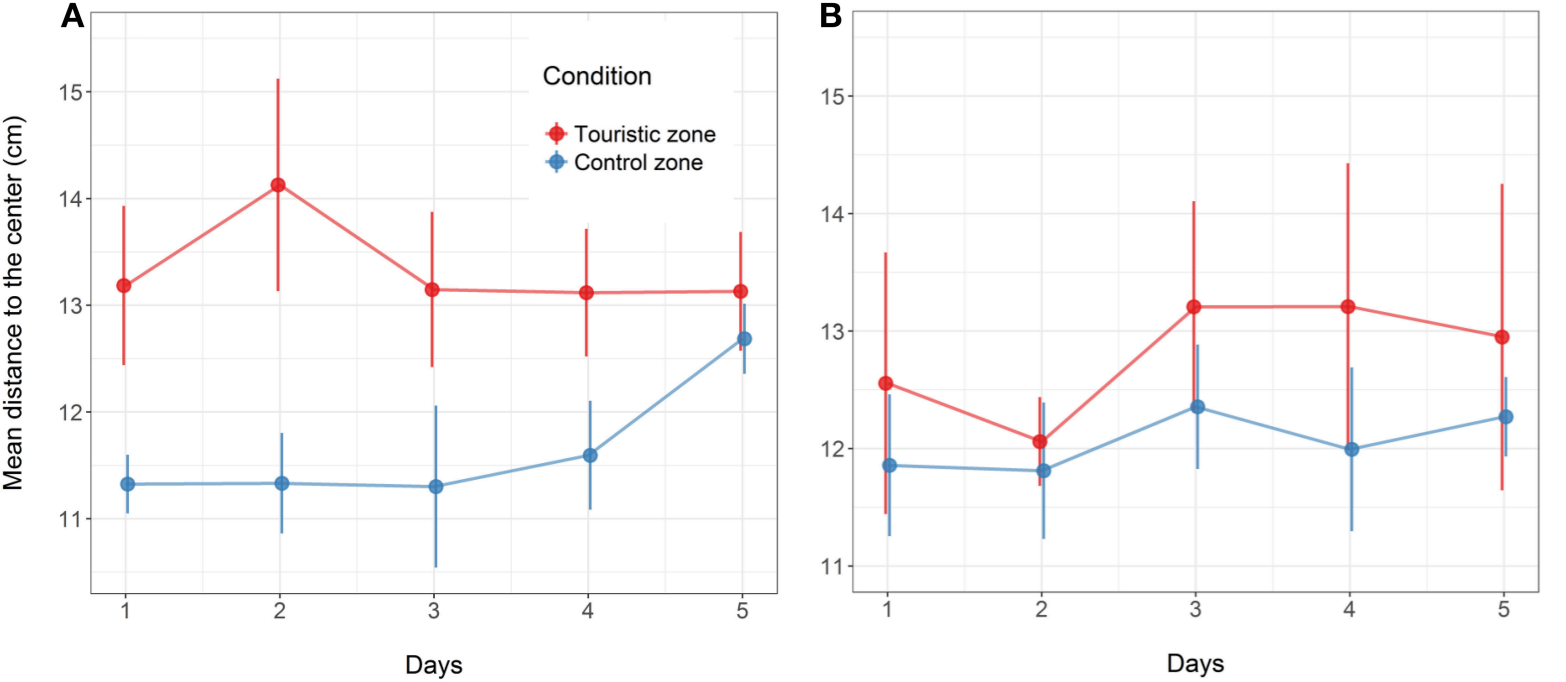
Mean distance to the center (cm) of the aquarium for all fish (each fish position evaluated at each image) throughout the 10 min before the artificial stress for the fish from the control or touristic zones. Values are given at the five experimental days in the two replicates of the study in June **(A)** and August **(B)**. Mean and standard error are given (*n* = 4 aquarium in June, *n* = 6 aquarium in August, with 17 fish per aquarium).

Following the mechanical stressor, fish responded by transiently increasing their swimming activity, similarly to a flight response (Figure 8). This was followed by a sudden decrease in activity until the fish almost stop moving, considered as a “freezing” response, more pronounced in fish from the tourism zone compared to control fish (*t*-value: −2.5, *p* < 0.02; Figures 7, 8, Table 4). The time needed to reach freezing tended to be longer for fish from the tourism zone when considering both sampling periods (*t*-value: 2.04, *p* = 0.056, Table 5) and significantly longer in June (*t*-value: 2.9, *p* < 0.01, see Figure 8A, Table 5). These differences were due to fish sampled in June in which both, freezing intensity and time to freeze, were respectively lower [model (ii), Table 4, Figure 7A] and longer [model (ii), Table 5, Figure 7A] in tourism compared to control zone fish. A similar pattern was observed in August (Figure 7B), although it was not significant [model (iii), Tables 4, 5]. Concerning thigmotaxis, tourism zone fish tended (*t*-value: 1.86, p: 0.08) to stay closer to the aquarium border when compared to control zone fish, during the 10 min before stress [model (i), Figure 9, Table 6]. As for time to freeze, this was mainly due to fish sampled in June, where differences in space use between tourism and control zone fish were significant [model (ii), Figure 9A, Table 6], while this was not significant in August [model (iii), Figure 9B, Table 6].

**Table 4:** Outputs of different linear mixed model testing the effects of time and zone (touristic or control) on freezing intensity for both trial [model (i)] and at each trial [model (ii), model (iii)].

**Model**	**Freezing Intensity** ~ **Zone** + **Time** + **(1|Trial/Aquarium)**					
**(i) June** + **August**	**Random effects**					
		**Groups**	**Name**	**Variance**	**Std. Dev**.	***n***
		Aquarium:Trial	(lntercept)	249.8	15.8	20
		SES	(lntercept)	537.7	23.2	2
		Residual		241.4	15.5	
		Number of observations: 100				
	**Fixed effects**					
		**Estimate**	**Std error**	***t-*****value**	***p*****-value**	
	(Intercept)	107.2	17.61	6.1	0.077	.
	Zone:Touristic	−19.4	7.7	−2.5	0.02	^*^
	Time	−0.9	1.1	−0.8	0.4	
	**Freezing Intensity** ~ **Zone** + **Time** + **(1|Aquarium)**					
**(ii) June**	**Random effects**					
		**Groups**	**Name**	**Variance**	**Std. Dev**.	***n***
		Aquarium	(Intercept)	78.24	8.845	8
		Residual		129.83	11.394	
		Number of observations: 40				
	**Fixed effects**					
		**Estimate**	**Std error**	***t*****-value**	***p*****-value**	
	(Intercept)	94.18	6.4	14.8	<0.0001	^***^
	Zone:Touristic	−22.3	7.2	−3.1	0.02	^*^
	Time	−1.75	1.3	−1.38	0.18	
	**Freezing Intensity** ~ **Zone** + **Time** + **(1|Aquarium)**					
**(iii) August**	**Random effects**					
		**Groups**	**Name**	**Variance**	**Std. Dev**.	***n***
		Aquarium	(Intercept)	377.8	19.4	12
		Residual		318.2	17.8	
		Number of observation: 60				
	**Fixed effects**					
		**Estimate**	**Std error**	***t*****-value**	***p*****-value**	
	(Intercept)	121.2	9.87	12.28	<0.0001	^***^
	Zone:Touristic	−17.43	12.13	−1.44	0.18	
	Time	−0.34	1.63	−0.21	0.84	

*Models with additive terms (zone + time) and interaction between terms (zone x time) were first compared using the Akaike's Information Criterion (AIC) so that the most parsimonious model was conserved*.

*** *< 0.001*,

* *< 0.05*.

**Table 5:** Outputs of different linear mixed model testing the effects of time and zone (touristic or control) on time to freeze, for both trial [model (i)] and at each trial [model (ii), model (iii)].

**Model**	**Time to freeze** ~ **Zone** + **Time** + **(Time|Trial/Aquarium)**					
**(i) June** + **August**	**Random effects**					
		**Groups**	**Name**	**Variance**	**Std. Dev**.	***n***
		Aquarium:Trial	(Ilntercept)	0.067	0.26	20
		Time	(Slope)	0.006	0.08	2
		Trial	(Intercept)	0.002	0.05	
		Time	(Slope)	5.83	2.4	
		Residual		54.14	7.36	
		Number of observations: 100				
	**Fixed effects**					
		**Estimate**	**Std error**	***t*****-value**	***p*****-value**	
	(Intercept)	94.79	1.88	50.4	<0.0001	^***^
	Zone:Touristic	3.04	1.49	2.04	0.056	.
	Time	−2.08	1.79	−1.17	0.45	
	**Time to freeze** ~ **Zone** + **Time** + **(1|Aquarium)**					
**(ii) June**	**Random effects**					
		**Groups**	**Name**	**Variance**	**Std. Dev**.	***n***
		Aquarium	(Intercept)	0	0	8
		Time	(Slope)	55.2	7.43	
		Number of observations: 40				
	**Fixed effects**					
		**Estimate**	**Std error**	***t*****-value**	***p*****-value**	
	(Intercept)	92.94	2.99	31	<0.0001	^***^
	Zone:Touristic	6.8	2.35	2.9	0.006	^**^
	Time	−3.8	0.83	−4.6	<0.0001	^***^
	**Time to freeze** ~ **Zone** + **Time** + **(1|Aquarium)**					
**(iii) August**	**Random effects**					
		**Groups**	**Name**	**Variance**	**Std. Dev**.	***n***
		Aquarium	(Intercept)	0	0	12
		Residual		51.7	71.9	
		Number of observation: 60				
	**Fixed effects**					
		**Estimate**	**Std error**	***t*****-value**	***p*****-value**	
	(Intercept)	96	2.37	40.6	<0.0001	^***^
	Zone:Touristic	0.53	1.86	0.29	0.78	
	Time	−0.37	0.66	−0.56	0.58	

*Models with additive terms (zone + time) and interaction between terms (zone x time) were first compared using the Akaike's Information Criterion (AIC) so that the most parsimonious model was conserved*.

*** *< 0.001*,

** *< 0.01*.

**Table 6:** Outputs of different linear mixed model testing the effects of time and zone (touristic or control) on the distance to the center (thigmotaxis), for both trial [model (i)] and at each trial [model (ii), model (iii)].

**Model**	**Distance to Center** ~ **Zone** + **Time** + **(1|Trial/Aquarium)**					
**(i) June** + **August**	**Random effects**					
		**Groups**	**Name**	**Variance**	**Std. Dev**.	***n***
		Aquarium:Trial	(Ilntercept)	1.53	1.24	20
		SES	(Intercept)	0	0	2
		Residual		1.42	1.19	
		Number of observations: 100				
	**Fixed effects**					
		**Estimate**	**Std error**	***t*****-value**	***p*****-value**	
	(Intercept)	11.52	0.49	23.27	<0.0001	^***^
	Zone:Touristi	1.12	0.6	1.86	0.08	.
	Time	0.13	0.08	1.49	0.14	
	**Distance to Center** ~ **Zone** + **Time** + **(1|Aquarium)**					
**(ii) June**	**Random effects**					
		**Groups**	**Name**	**Variance**	**Std. Dev**.	***n***
		Aquarium	(Intercept)	0.36	0.6	8
		Time	(Slope)	1.22	1.1	
		Number of observations: 40				
	**Fixed effects**					
		**Estimate**	**Std error**	***t*****-value**	***p*****-value**	
	(Intercept)	11.37	0.54	21.19	<0.0001	^***^
	Zone:Tourist	1.69	0.55	3.09	0.02	^*^
	Time	0.09	0.12	0.76	0.45	
	**Distance to Center** ~ **Zone** + **Time** + **(1|Aquarium)**					
**(iii) August**	**Random effects**					
		**Groups**	**Name**	**Variance**	**Std. Dev**.	***n***
		Aquarium	(Intercept)	2.47	1.57	12
		Residual		1.58	1.26	
		Number of observation: 60				
	**Fixed effects**					
		**Estimate**	**Std error**	***t*****-value**	***p*****-value**	
	(Intercept)	11.61	0.76	15.22	<0.0001	^***^
	Zone:Tourist	0.74	0.96	0.77	0.46	
	Time	0.15	0.11	1.28	0.21	

*Models with additive terms (zone + time) and interaction between terms (zone x time) were first compared using the Akaike's Information Criterion (AIC) so that the most parsimonious model was conserved*.

*** *< 0.001*,

* *< 0.05*.

## Discussion

Using the tetra fish to represent continuous exposition to humans, we demonstrated that the expression of genes related to the stress axis and neurogenesis were modulated by contact with tourists. Similarly, differences in basal cortisol values, behavior and stress sensitivity persisted in a neutral artificial environment, with similar densities, even 12 days after being extracted from their environment. Since fish densities were different between the two natural sites, it is however not clear whether the described effects are a direct or an indirect consequence of ecotourism. In any case, our findings highlight the need to evaluate the impacts of ecotourism at multiple levels.

First, an increase in stress signaling pathway receptors (GR and MR) was observed in fish from the tourism zone, suggesting that fish from the tourism zone are more sensitive to corticosteroids than fish from the control site. These patterns are opposed to those usually observed in chronically stressed animals (Lupien et al., 2009) but, on the contrary, similar to those described in animals chronically exposed to “non-threatening” stimuli, as observed in enriched environments. For instance, early and long-term exposure to sensory and occupational enrichment are considered to stimulate the brain and strongly modify the HPI regulation system (Näslund and Johnsson, 2016), mainly by increasing expression of glucocorticoid receptors (Sampedro-Piquero et al., 2014). Additionally, animals grown in enriched environments usually also exhibit increased neurogenesis (van Praag et al., 2000), flooded by increased brain expression of genes such as *neuroD* and *pcna* (von Krogh et al., 2010; Salvanes et al., 2013). Increased *neuroD* expression, but not *pcna* expression, was also observed in fish from the tourism zone. Therefore, gene expression data, describing enhanced expression of cortisol receptors and one gene related to neurogenesis in fish sampled in the tourism zone, advocate for describing the tourism zone as a stimulating environment, analogous to an enriched environment.

In our study, the basal cortisol level was higher in fish from the tourism zone compared to fish from the control zone. Although this is the first study evaluating change in basal cortisol following tourism exposure in fish, these results are consistent with studies on mammals (Creel et al., 2013; Maréchal et al., 2016), but not birds and iguanas (Müllner et al., 2004; Walker et al., 2006; Ellenberg et al., 2007; French et al., 2010; Villanueva et al., 2011; Soldatini et al., 2015). Hence, although tourism exposure intensity and food provisioning might affect the final result, a taxonomic effect might also account for the differential results observed. The reason for the discrepancy between taxa is not yet clear and this calls for more research.

Following the artificial stressor, tetras from the tourism zone also presented a steeper increase in cortisol production, compared to control fish. Although this stressful event (tapping iron bar decorated with cloth on the aquarium) was not necessarily representative of a touristic stress, it represents an unpredictable event that could happen in natural conditions. The higher cortisol production we report is consistent with several studies describing a hyper-sensitivity of the HPA/HPI axis following an artificial stress in animals repeatedly disturbed by humans (Müllner et al., 2004; Walker et al., 2005; Ellenberg et al., 2007; French et al., 2010; Lima et al., 2014; Soldatini et al., 2015) including fishes (Lima et al., 2014), although there are exceptions (Romero, 2002; Walker et al., 2006).

In terms of behavior, fish from the tourism zone were more prone to thigmotaxis and freezing response following the unpredictable stressor. Thigmotaxis has been previously triggered by high social density (Shams et al., 2015), sound (Shafiei Sabet et al., 2016) and chemical cues (Nema et al., 2016). In addition, freezing bouts are a well-established result of the HPI axis activation (Galhardo and Oliveira, 2009), being related to stressors such as anthropogenic noise (Slabbekoorn, 2013). These behaviors are indeed common stress responses, suggesting that fish chronically exposed to tourism tend to be, on average, more stressed in a novel environment and to be more sensitive to an acute stressor.

High HPI activity, high thigmotaxis propensity and enhanced freezing response, as observed in fish from the tourism zone, is commonly described as a reactive coping style (Koolhaas et al., 1999), as opposed to proactive coping style. In zebrafish, reactive individuals also display increased basal cortisol concentrations and higher cortisol secretion over time following a stress (Tudorache et al., 2013), together with high *mr* gene expression (Bos et al., 2017). Interestingly, reactive fishes were also recently shown to present high level of *pcna* and *neurod* in other contexts (Bos et al., 2017; Vindas et al., 2017), concordant with our data on gene expression and are generally characterized by high cognitive and learning capacities (Sih and Giudice, 2012; Podgorniak et al., 2016). Contrarily, proactive individuals showing inflexible behavior and less fear are generally associated with limited neural plasticity (Øverli and Sørensen, 2016; Vindas et al., 2017). Overall, our results suggest that chronic exposure to tourism leads to a mean population shift in coping style by enhancing reactive phenotypes, with higher level of cortisol, *neurod, mr, gr* (marginal significance value) and higher freezing intensity and anxiousness. From an evolutionary standpoint, it has been proposed that this specific response: reactive individuals are favored under chronic stress and/or unpredictable circumstances (e.g., tourism), whereas proactive coping styles may be of greater advantage in low-stress and/or predictable environments, would be adaptive (Sørensen et al., 2013). It is then possible that the phenotypic swift observed here occurs without further changes at the population level. Nevertheless, if this shift of coping style results from adaptation to local conditions, then it could impose strong energetic costs, latter transduced at the population scale (reviewed in Geffroy et al., 2017). Although not directly tested here, other studies showed that a modification of coping style would affect dispersion processes (Wey et al., 2015), brood size (Nicolaus et al., 2015) and ultimately, global population fitness (Réale et al., 2007). Yet, no dramatic events such as abandonment of the site, reduced reproduction or increased mortality were noticed. Rather, the focal fish appeared to thrive around humans, as indicated by the higher fish density observed in the TZ in August and September. Many explanations could be invoked; particularly food provisioning (intendedly or unintendedly through deadskin) or artificial protection against predators, also referred as human shield effect (Geffroy et al., 2015). Owing to the specific life history traits of this Tetra species, that also performs mutilating predation alone or in groups (*sensu* Lima et al., 2012) by feeding on other fishes' body parts (scales, fins, mucus and epidermis, Lima et al., 2012), high density could readily becomes problematic for the community (Balduino et al., 2017). The continuous monitoring of this site in the future would be an excellent opportunity to evaluate accurately the above-described possible causes and consequences.

Overall, our findings support the hypothesis that nature-based tourism has lasting physiological and behavioral effects. Both human presence *per se* and food provisioning in natural areas may affect coping styles and possibly population dynamic. The effect of continuous contacts with humans in the wild have been overlooked and our study suggests that a progressive shift in coping style is the first likely outcome that would be observed in other species, if the exponential interest for nature-based tourism continues that way.

## Data accessibility

All data are available as additional files. Both, fish tracking algorithm and results are available on the permanent link: https://bioimagerie.univ-rennes1.fr/publi/geffroy.html.

## Author contributions

BG and EB conceived the idea and designed the experiment. BG, AB, SP, MG-R, RM, MM, and BS conducted the analysis. J-PB, LN, and EB participated in data collection and project management. BG, J-PB, BS, and EB wrote the manuscript with insights from all authors.

### Conflict of interest statement

The authors declare that the research was conducted in the absence of any commercial or financial relationships that could be construed as a potential conflict of interest.
